# *Amblyceps waikhomi*, a New Species of Catfish (Siluriformes: Amblycipitidae) from the Brahmaputra Drainage of Arunachal Pradesh, India

**DOI:** 10.1371/journal.pone.0147283

**Published:** 2016-02-03

**Authors:** Achom Darshan, Akash Kachari, Rashmi Dutta, Arijit Ganguly, Debangshu Narayan Das

**Affiliations:** 1 Center with Potential for Excellence in Biodiversity, Rajiv Gandhi University, Rono Hills, Doimukh, India; 2 Fishery and Aquatic Ecology Laboratory, Department of Zoology, Rajiv Gandhi University, Rono Hills, Doimukh, India; 3 Department of Zoology, Achhruram Memorial College, Jhalda, Purulia, India; University of Innsbruck, AUSTRIA

## Abstract

*Amblyceps waikhomi* sp. nov. is described from the Nongkon stream which drains into the Noa Dehing River, a tributary of the Brahmaputra River, in Arunachal Pradesh, India. The new species can be distinguished from congeners (except *A*. *torrentis*) in having a deeper body depth at anus. It further differs from congeners (except *A*. *mangois* and *A*. *serratum*) in having fewer vertebrae, from *A*. *mangois* in lacking (vs. having) strongly-developed projections on the proximal lepidotrichia of the median caudal-fin rays, and in having a longer, wider, and deeper head; and from *A*. *serratum* in having a posteriorly smooth (vs. with 4–5 serrations) pectoral spine, and unequal jaw length (lower jaw longer and weakly-projecting anteriorly vs. equal upper and lower jaws). It additionally differs from *A*. *murraystuarti*, *A*. *torrentis*, *A*. *apangi*, *A*. *laticeps*, and *A*. *cerinum* in having a deeply forked (vs. emarginate or truncate) caudal fin. This species is the seventh amblycipitid species known to occur in the Ganga-Brahmaputra River system.

## Introduction

Fishes of the genus *Amblyceps* Blyth are small-bodied, elongate catfishes, occurring in fast flowing streams and rivers of mainland Southeast Asia and the Indian subcontinent [1]. The genus can be diagnosed by a double fold of skin on both the upper and lower lips, pinnate-like rays on the anterior margins of the procurrent caudal-fin rays, the anterior nostril situated immediately anterior to the base of the nasal barbel, and the epiphyseal commissure of the supraorbital sensory canals located immediately anterior to and not passing through the epiphyseal bar [2].

Eighteen species of *Amblyceps* are presently considered valid *viz*. *A*. *apangi* Nath and Dey, *A*. *arunachalensis* Nath and Dey, *A*. *caecutiens* Blyth, *A*. *carinatum* Ng, *A*. *cerinum* Ng and Wright, *A*. *foratum* Ng and Kottelat, *A*. *kurzii* (Day), *A*. *laticeps* (McClelland), *A*. *macropterus* Ng, *A*. *mangois* (Hamilton), *A*. *murraystuarti* Chaudhuri, *A*. *platycephalus* Ng and Kottelat, *A*. *protentum* Ng and Wright, *A*. *serratum* Ng and Kottelat, *A*. *tenuispinis* Blyth, *A*. *torrentis* Linthoingambi and Vishwanath, *A*. *tuberculatum* Linthoingambi and Vishwanath, and *A*. *variegatum* Ng and Kottelat [3].

During recent ichthyological surveys of the eastern part of Arunachal Pradesh, five specimens of *Amblycep*s were collected from a stream flowing into Noa Dehing River of the upper Brahmaputra basin in Namsai District. After a detailed comparison with all 18 congeners, we concluded that they represent an unnamed *Amblyceps* species, which is herein described as *Amblyceps waikhomi* sp. nov.

## Materials and Methods

Measurements were made on the left side of specimens with a digital caliper to the nearest 0.1 mm and fin rays were counted under a Nikon SMZ800 stereo-zoom microscope. Morphometric measurement and fin ray counts followed Ng and Wright [4]. Measurements of head length (HL) and body parts were expressed as percent proportions of standard length (SL) while measurements of the subunits of the head were expressed as percent proportions of head length. Counts of gill rakers and vertebrae followed Roberts [5] and Roberts [6] respectively. Clearing and staining of specimens followed Hollister [7]. Nomenclature of the bone followed Chen and Lundberg [2]. Procurrent rays of the caudal fin were counted from anterior to posterior separately for the upper and lower lobe. The type specimens were deposited in the Rajiv Gandhi University Museum of Fishes (RGUMF), Arunachal Pradesh and also in the Zoological Survey of India, Arunachal Pradesh Regional Centre (ZSI/APRC), Itanagar.

The water quality of the habitat was analysed by taking three replicates randomly from the type locality of the new species during January 2014. Estimation of dissolved carbon dioxide (DCO_2_), alkalinity, and hardness followed APHA [8]. Temperature, pH, conductivity, and dissolve oxygen (DO) were measured using a Systronics Water Analyser 321 (Systronics, India). Water current and transparency were assessed using the JDC flowatch kit (JDC Instruments, Switzerland) and Secchi disk respectively.

We followed the rules of the Rajiv Gandhi University Institutional Animal Ethical Committee, Arunachal Pradesh, and the work was approved by the committee and the present work did not involve any endangered species or protected areas.

### Nomenclatural Acts

The electronic edition of this article conforms to the requirements of the amended International Code of Zoological Nomenclature, and hence the new names contained herein are available under that Code from the electronic edition of this article. This published work and the nomenclatural acts it contains have been registered in ZooBank, the online registration system for the ICZN. The ZooBank LSIDs (Life Science Identifiers) can be resolved and the associated information viewed through any standard web browser by appending the LSID to the prefix "http://zoobank.org/". The LSID for this publication is: urn:lsid:zoobank.org:pub: 96B8624A-2A28-401F-BD79-366FB110435C. The electronic edition of this work was published in a journal with an ISSN, and has been archived and is available from digital repositories of PubMed Central and LOCKSS.

## Results

*Amblyceps waikhomi* sp. nov.

urn:lsid:zoobank.org:act:8F9219B2-619E-4321-8C2E-F31926DAD0FF

### Type specimens

Holotype. ZSI/APRC/P-1125, 42.9 mm SL; India, Arunachal Pradesh, Namsai District, Nongkon stream at Nongkon village draining into Noa Dehing River (Brahmaputra basin), 27°36’05”N, 95°50’51”E; Akash Kachari, 5 October 2013.

Paratypes. Locality and collector as for holotype, RGUMF 269, 40.2 mm SL, 1 cleared and stained (c&s), 20 June 2014; RGUMF 270, 37.4 mm SL, 1 c&s, 12 October 2014; RGUMF 271, 30.4–44.7 mm SL, 2, 6 December 2014.

### Diagnosis

**Fig 1:**
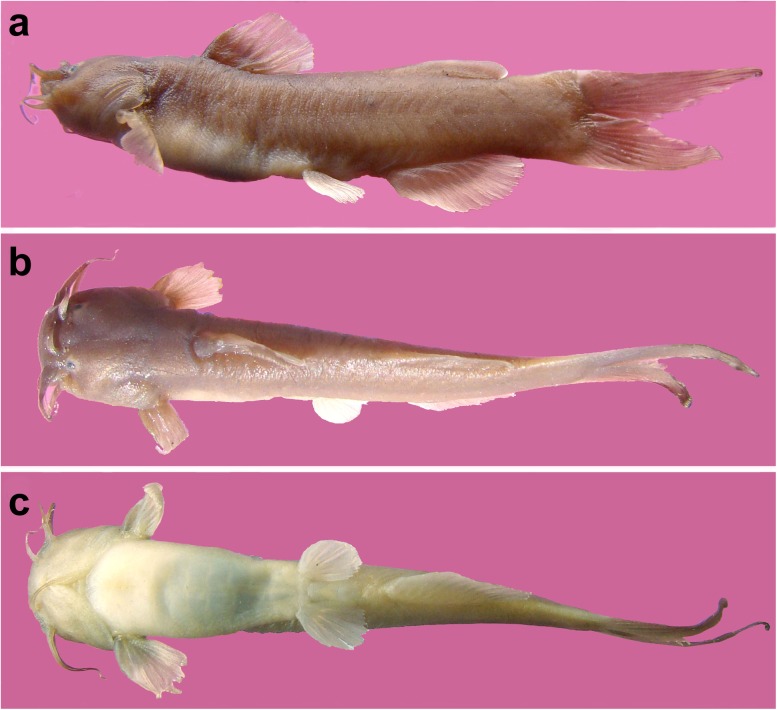
*Amblyceps waikhomi*, holotype, ZSI/APRC/P-1125, 42.9 mm SL: a. lateral b. dorsal c. ventral views.

*Amblyceps waikhomi* sp. nov.(Fig 1, S1 Fig) differs from all congeners in having a deeper body (depth at anus 17.0–20.3% SL vs. 7.6–16.9) and fewer (except *A*. *mangois* and *A*. *serratum*) total vertebrae (34–35 vs. 37–48) (S1 Table). It differs from *A*. *mangois* in lacking (vs. having) strongly-developed projections on the proximal lepidotrichia of the median caudal-fin rays and in having a longer, wider, and deeper head (S2 Table) (length: 22.1–24.3% SL vs. 18.8–21.3, width: 20.0–21.9% SL vs. 17.1–18.3, depth: 13.9–18.0% SL vs. 11.6–13.2); and from *A*. *serratum* in having a posteriorly smooth (vs. with 4–5 serrations) pectoral spine, and unequal jaw length (lower jaw longer and weakly-projecting anteriorly vs. equal upper and lower jaws). It additionally differs from *A*. *murraystuarti*, *A*. *torrentis*, *A*. *apangi*, *A*. *laticeps*, and *A*. *cerinum* in having a deeply forked (vs. emarginate or truncate) caudal fin.

### Description

Morphometric data are shown in Table 1. Body short, stout, laterally compressed. Dorsal profile rising evenly from tip of snout to dorsal-fin origin, then straight upto middle of adipose-fin base, thereafter gently sloping ventrad to the end of caudal peduncle. Ventral profile convex up to anal opening, then gently sloping dorsally up to end of anal-fin base, thereafter gently sloping ventrad towards caudal-fin base. Anus and urogenital openings located slightly anterior to anal-fin origin.

**Table 1:** Morphometric data of *Amblyceps waikhomi* sp. nov. (n = 5).

	Holotype	Range	Mean±SD
Total length	61.8	39.7–61.8	-
Standard length (SL)	42.9	30.4–44.7	-
In % SL			
Predorsal length	27.7	25.7–30.6	28.2±2.05
Preanal length	64.1	58.2–64.1	61.6±2.96
Prepelvic length	50.3	48.3–50.3	49.5±0.90
Prepectoral length	20.2	19.2–23	20.9±1.61
Length of dorsal-fin base	11.6	10.1–14.5	12.1±1.83
Length of anal-fin base	18.4	16.7–19.6	18.2±1.20
Pelvic-fin length	12.3	9.6–12.3	11.1±1.13
Pectoral-fin length	17.0	16.8–19.0	17.5±1.04
Upper lobe of caudal-fin length	46.6	27.7–46.6	34.5±8.41
Lower lobe of caudal-fin length	31.4	25.5–31.4	27.6±2.64
Length of adipose-fin base	23.7	20.3–23.7	22.0±1.40
Dorsal to adipose distance	17.0	17.0–21.3	19.8±1.92
Length of caudal peduncle	18.6	15.5–18.6	17.5±1.44
Depth of caudal peduncle	13.0	13.0–16.4	14.3±1.48
Body depth at anus	17.0	17.0–20.3	18.3±1.58
Head length	23.0	22.1–24.3	23.3±0.95
Head width	20.0	20.0–21.9	20.7±0.91
Head depth	17.0	13.9–18.0	15.9±1.92
In % HL			
Snout length	27.2	23.4–27.2	25.1±1.63
Eye diameter	7.0	6.7–7.5	7.1±0.35
Inter orbital distance	39.3	21.6–43.4	36.0±9.78
Nasal barbel length	78.7	71.6–90.3	79.5±7.84
Maxillary barbel length	105.5	90.5–114.4	101.4±10.67
Inner mandibular barbel length	78.7	68.0–78.7	72.0±4.65
Outer mandibular barbel length	86.8	78.3–86.8	81.8±4.21

Head depressed. Snout rounded. Mouth terminal with unequal jaws, lower slightly longer; lips papillate, with double fold of thickened skin. Premaxillary tooth band semicircular, bearing short, conical, posteriorly directed teeth. Mandibular teeth short, conical, arranged in narrow crescentic band. Eye small, rounded, and subcutaneous. Anterior nostril short, tubular, situated immediately anterior to base of nasal barbel. Nasal barbel extending beyond upper margin of upper gill opening, not reaching posterior margin of opercle. Maxillary and outer mandibular barbels reaching to base of last pectoral-fin ray. Inner mandibular barbel extending to base of pectoral-fin. Skin on head and body tuberculate. Lateral line incomplete, curved downward, and terminating at a point slightly anterior to vertical through dorsal-fin origin. First branchial arch with 2+7 (n = 5) gill rakers. Gill membranes narrowly joined at isthmus, with 10 (n = 2) branchiostegal rays.

Dorsal fin with a spinelet, a spine, and 6 (n = 5) branched rays; its origin closer to snout tip than to adipose-fin origin; posterior margin of fin convex; fin base fleshy and swollen. Dorsal-fin spine smooth, short and straight, distal tip sharply pointed, its length reaching one-third of fin height. Adipose fin short, low, commencing from vertical midway between anus and anal-fin origin, posterior margin well separated from caudal fin. Pectoral fin with a smooth spine and 6 (n = 5) branched rays; origin anterior to vertical through posterior margin of operculum posterior margin convex. Pectoral-fin spine longer than dorsal-fin spine, straight, anterior and posterior margins smooth. Skin covering pectoral-fin base and skin covering spine swollen. Pelvic fin with i–ii,4–5 rays, tip of adpressed fin reaching beyond urogenital opening but not anal-fin origin. Anal fin with iii,10 rays. Caudal fin deeply forked with i,7,8,i (n = 5) principal rays, simple-principal and segmented procurrent rays of upper and lower lobe bears pinnate like rays anteriorly, upper lobe longer than lower (S2 Fig).

#### Coloration

In 70% ethanol: Dorsal and lateral surfaces of head and body brownish, ventrally creamy.

#### Distribution

**Fig 2:**
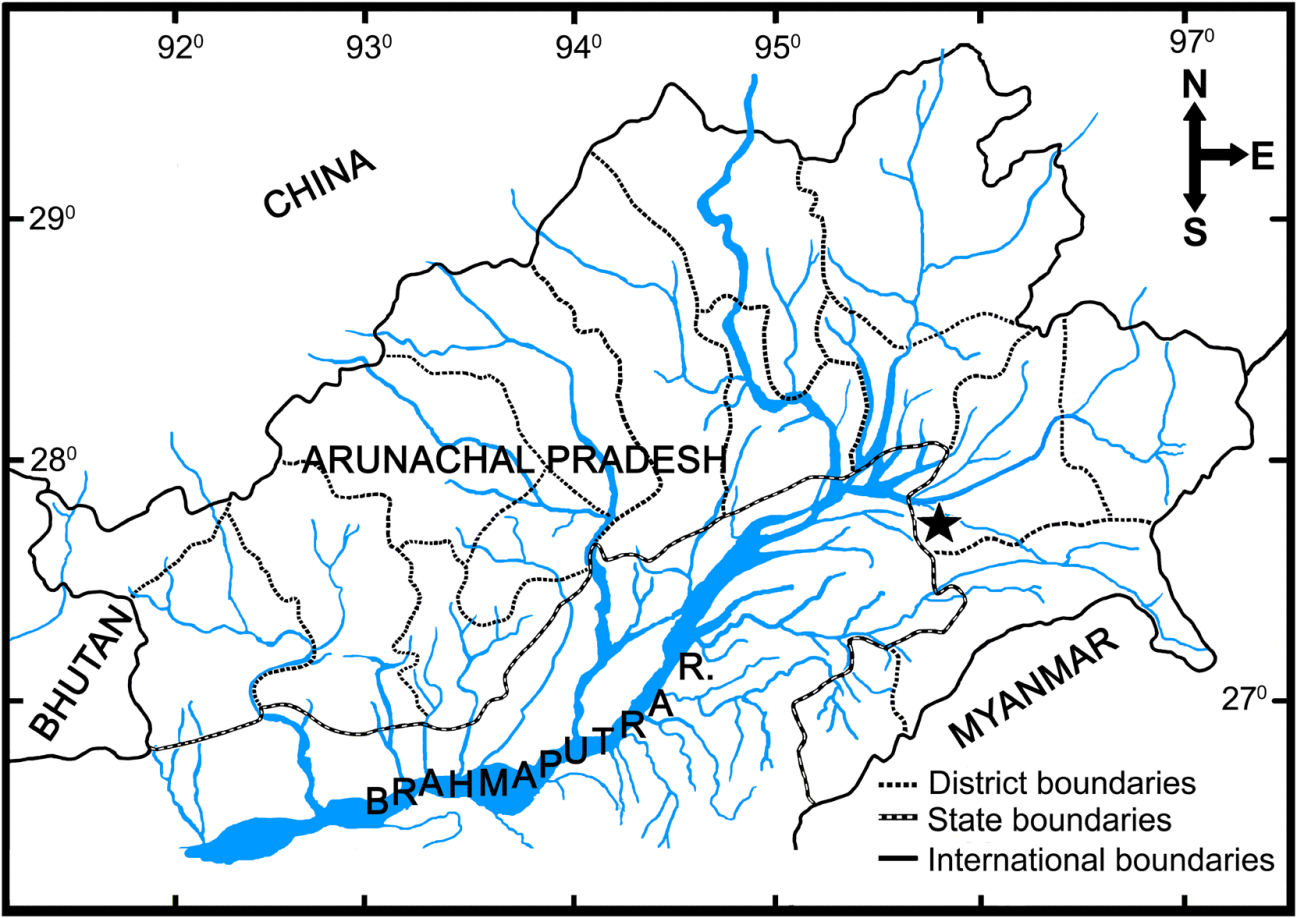
Map showing type locality of *Amblyceps waikhomi* (star marked).

Presently known only from its type locality, Nongkon stream draining to Noa Dehing River (Brahmaputra basin), Namsai District, Arunachal Pradesh (Fig 2).

#### Habitat

**Fig 3:**
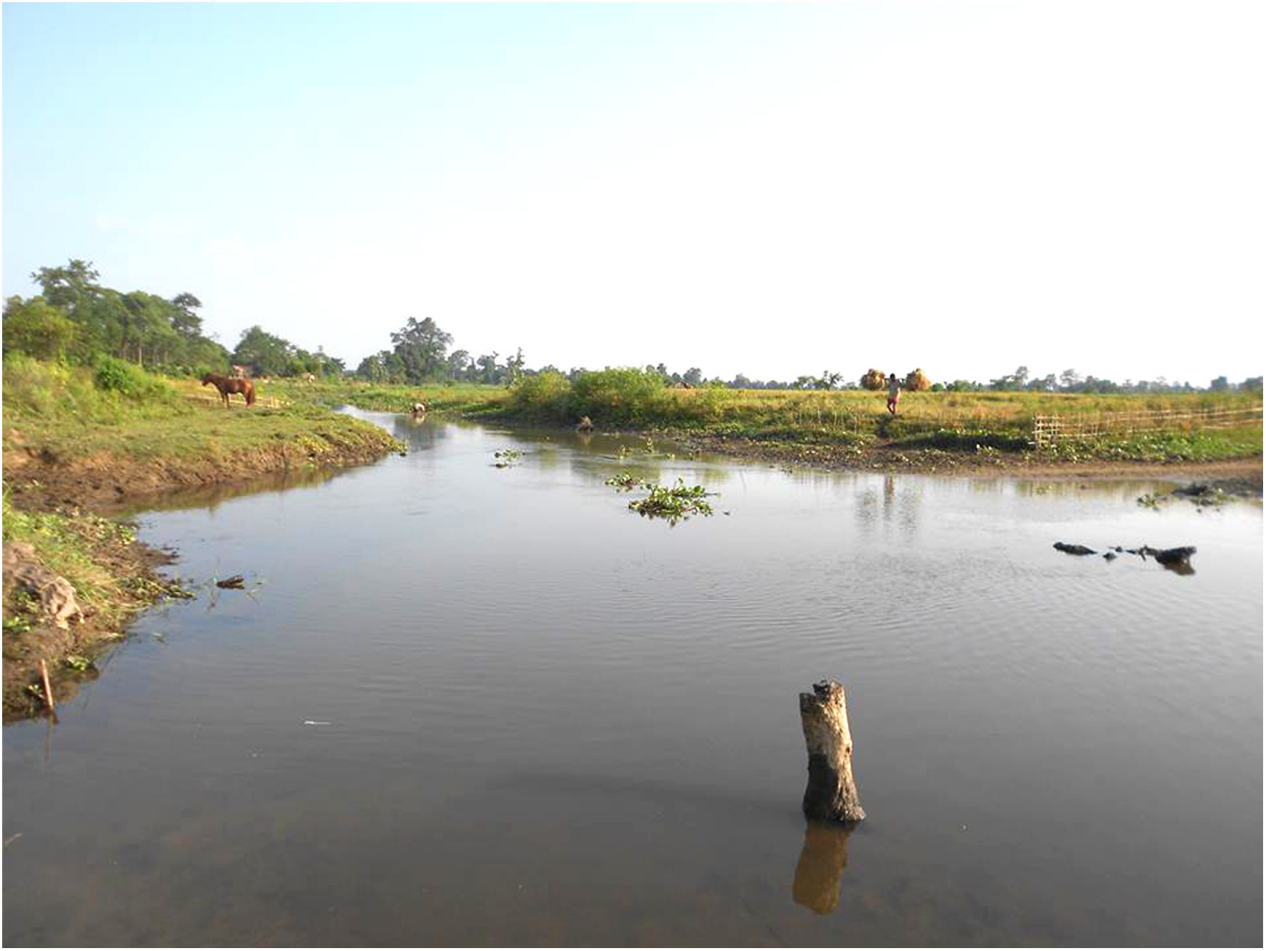
Type locality of *Amblyceps waikhomi*, Nongkon stream in Namsai district, Arunachal Pradesh.

The new species was collected from a slow moving stream (water current 0.16 m/s) with a bottom substrate dominated by sand, occasionally associated with mud (Fig 3). The species was often encountered under submerged logs and bamboo. Water hyacinth was the dominant macrophyte of the stream. Chemical parameters of the stream were DO 6.75 mg/l, DCO_2_ 1.53 mg/l, alkalinity 66.06 mg/l, and hardness 71.8 ± 3.05 mg/l; while the physical parameters were pH 6.78, air temperature 23.8 ±0.87°C, water temperature 23.5 ±0.96°C, transparency 79.5 ±1.93 cm,and conductivity 173.33 μS.

#### Etymology

The new species is named after Waikhom Vishwanath, honouring his outstanding contribution to freshwater ichthyology in the Indian subcontinent.

## Discussion

**Fig 4:**
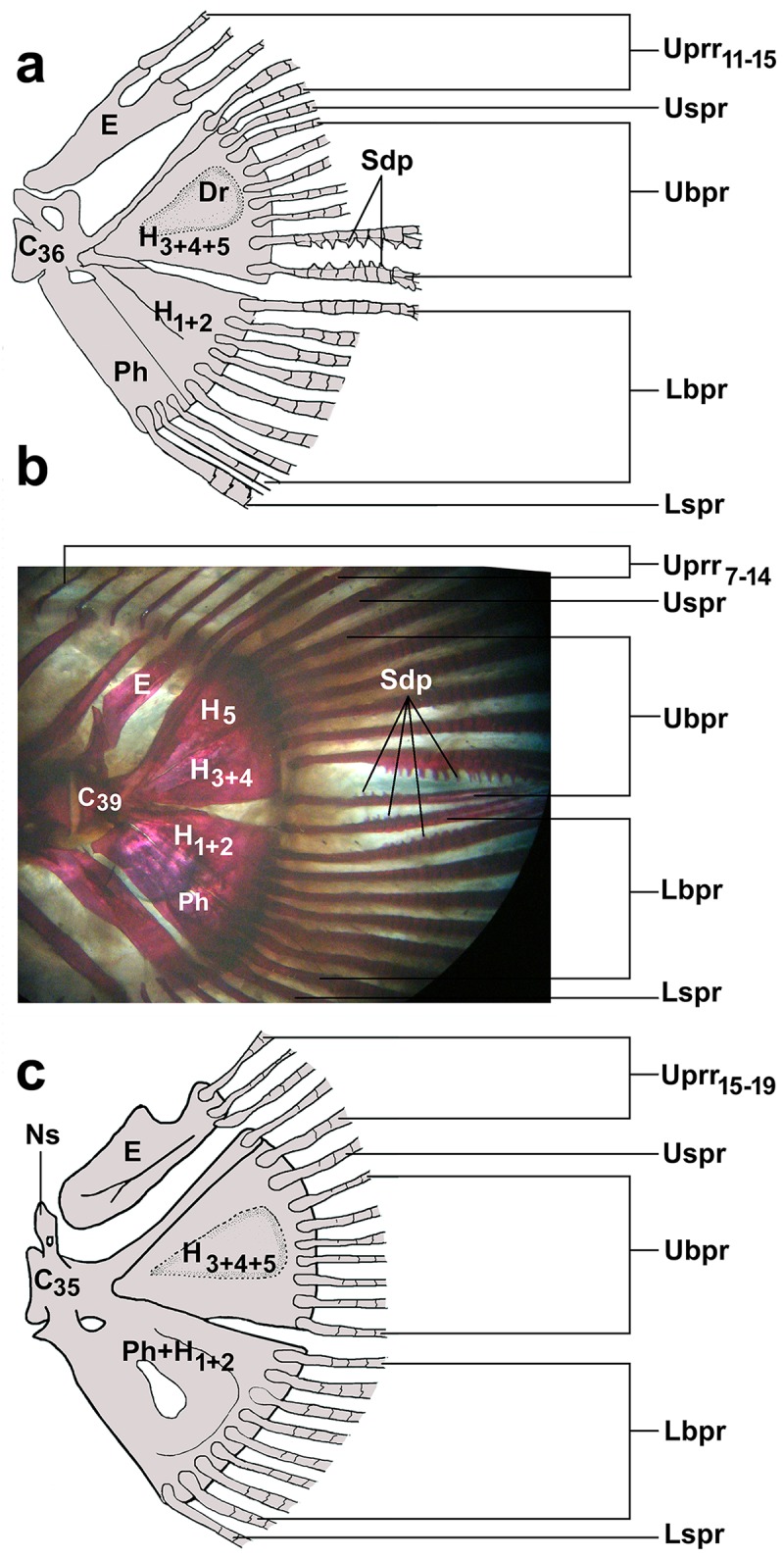
Caudal fin. a. *A*. *mangois* (RGUMF unregistered, 45.2 mm SL), b. *A*. *arunachalensis* (RGUMF 117, 85.1 mm SL), and c. *A*. *waikhomi* (RGUMF 269, 40.2 mm SL). (Parphypural may be separated from hypural in *A*. *waikhomi*, not shown in figure). [Ph: parhypural, H: hypural plate, Dr: depression, E: epural, C: centrum Uprr: upper procurrent ray, Uspr: upper simple principal ray, Ubpr: upper branched principal ray, Sdp: strongly developed-projections, Lbpr: lower branched principal ray, Lspr: lower simple principal ray]

*Amblyceps* species have unossified pinnate-like rays (4–5 pinnate rays per lepidotrichium) on the anterior margins of the procurrent caudal-fin rays [2]. In the present study, we examined cleared and stained specimens of *A*. *mangois*, *A*. *arunachalensis*, *A*. *apangi*, and *A*. *waikhomi* and found the pinnate like rays (except *A*. *apangi*) only on the distal half of the anterior margin of the segmented procurrent rays and unbranched principal rays. Ng and Kottelat [9] further reported the presence of pinnate like rays along the median caudal-fin rays of *Amblyceps* of the Indian subcontinent. This finding was confirmed by our observations on *A*. *mangois* (Fig 4a; S3 Fig) and *A*. *arunachalensis* (Fig 4b), which exhibited strongly-developed ossified projections on the proximal lepidotrichia of the median caudal-fin rays. However, this feature was absent in *A*. *waikhomi* (Fig 4c) and *A*. *apangi*. In *A*. *arunachalensis* these ossified projections were observed between the two lowermost branched principal rays of the upper lobe, between the two uppermost branched principal rays of the lower lobe, and also between the lowermost and the uppermost rays of the upper and lower lobe of the caudal fin respectively. In the case of *A*. *mangois*, these projections were located only between the two lowermost branched rays of the upper lobe of caudal fin.

With the description of *Amblyceps waikhomi*, seven species of *Amblyceps* are now known from the Ganga-Brahmaputra River system viz. *A*. *waikhomi*, *A*. *mangois*, *A*. *arunachalensis*, *A*. *tenuispinis*, *A*. *apangi*, *A*. *laticeps*, and *A*. *cerinum*. *Amblyceps* species can be divided into two groups: one having a deeply forked caudal fin, and the other with an emarginate or truncate caudal fin. In addition to the characters mentioned in the diagnosis (specifically for the congeners in the first group, to which *A*. *waikhomi* belongs), *A*. *waikhomi* further differs from *A*. *tenuispinis* in having a shorter snout (23.4–27.2% HL vs. 33.6–43.3) and dorsal to adipose distance (17.0–21.3% SL vs. 23.9–34.0), a longer pectoral fin (16.8–19.0% SL vs. 15.1–16.6), and a deeper caudal peduncle (13.0–16.4% SL vs. 9.6–12.9); from *A*. *arunachalensis* in having a longer predorsal (25.7–30.6% SL vs. 22.5–23.2), prepelvic (48.3–50.3% SL vs. 45.1–45.9), prepectoral (19.2–23.0% SL vs. 18.2–19.8), and adipose-fin base (20.3–23.7% SL vs. 18.1–19.8) lengths and fewer pleural ribs (7 vs. 12); and from *A*. *macropterus* in having fewer vertebrae (34–35 vs. 37), a shorter adipose-fin base (20.3–23.7% SL vs. 28.3), and in lacking (vs. having) the strongly-developed projections on the proximal lepidotrichia of the median caudal-fin rays.

*Amblyceps waikhomi* can be further distinguished from *A*. *carinatum* by its shorter adipose-fin base length (20.3–23.7% SL vs. 37.5–44.6) and longer dorsal to adipose distance (17.0–21.3% SL vs. 7.8–10.7); from *A*. *tuberculatum* by its shorter caudal peduncle length (15.5–18.6% SL vs. 21.2–22.4), shorter dorsal to adipose distance (17.0–21.3% SL vs. 27.8–28.0), and incomplete (vs. complete) lateral line; from *A*. *kurzii* by its longer adipose-fin base (20.3–23.7% SL vs. 15.1–18.3), shorter dorsal to adipose distance (17.0–21.3% SL vs. 30.1–30.6), and deeper caudal peduncle (13.0–16.4% SL vs. 9.8–10.7).

*Amblyceps waikhomi* can be further distinguished from *A*. *platycephalus* by its fewer principal caudal-fin rays (17 vs. 20); from *A*. *caecutiens* in having a larger eye (6.7–7.5% HL vs. 2.0–3.4) and shorter adipose-fin base (20.3–23.7% SL vs. 25.6–33.5); from *A*. *protentum* in having a longer prepelvic (48.3–50.3% SL vs. 42.8–47.8), prepectoral (19.2–23.0% SL vs. 15.9–18.3), and pectoral-fin (16.8–19.0% SL vs. 11.2–14.4) lengths, shorter and deeper caudal peduncle (length:15.5–18.6% SL vs. 20.0–25.6; depth: 13.0–16.4% SL vs. 8.0–10.3), shorter snout (23.4–27.2% HL vs. 30.1–34.6), and shorter dorsal to adipose distance (17.0–21.3% SL vs. 26.3–32.2); and from *A*. *variegatum* by its uniformly brownish (vs. mottled) body coloration.

### Comparative material

*Amblyceps mangois*: ZSI-NRS/F2556, 31,40.0–49.4 mm SL, India: Uttar Pradesh, Saharanpur, Padhoe River at Kalsia Ghat, Ganga River basin, K.P. Singh, 20 January 1972. RGUMF, unregistered, 3, 45.2–55.5 mm SL, India: Uttarakhand, Nainital district, Gola River at Kathgodam, Ganga River basin, A. Darshan, 30 April 2011. MUMF 14301–14302, 2, 35.5–37.1 mm SL, India: Barak River at Silchar (Assam), M. Shantakumar and K. Nebeshwar, 16 December 2000. MUMF 14061, 1, 48 mm SL, India: Arunachal Pradesh, Dikrong River, K. Nebeshwar.

*Amblyceps arunachalensis*: RGUMF 117, 82.6–97.2 mm SL, 3, India: Arunachal Pradesh, Subansiri River at Daporijo, Brahmaputra basin, 7 June 2005.

*Amblyceps apangi* (S4 Table): RGUMF 118, 160.0 mm SL, India, Arunachal Pradesh, Papum Pare district, Dikrong River at Sagalee, Brahmaputra basin, 7 January, 2005. RGUMF 114, 45–91.2 mm SL, 7, India, Arunachal Pradesh, Papum Pare district, Dikrong River, Brahmaputra basin, 17 July 2005. RGUMF 116, 71.5–120.0 mm SL, 7, India, Arunachal Pradesh, Subansiri River at Daporijo, Brahmaputra basin, 7 June 2005.

*A*. *torrentis*: MUMF 6170, 85.0 mm SL, holotype, India, Manipur, Ukhrul district, Laniye River at Jessami village, Chindwin basin. MUMF 2111, 1, 76.8 mm SL, paratype, India: Manipur, Ukhrul district, Challou River at Chingai village, Chindwin basin. Additional data from Linthoingambi and Vishwanath [10].

*A*. *tuberculatum*: MUMF 6184, holotype, 97.2 mm SL, India: Manipur, Chandel district, Lokchao River at Moreh town, Chindwin basin. MUMF 6179–6180, 69.4–76.3 mm SL, 2, paratype, same data as above. Additional data from Linthoingambi and Vishwanath [10].

*A*. *platycephalus*, *A*. *variegatum*, *A*. *foratum*, and *A*. *serratum*: Data from Ng and Kottelat [9].

*A*. *caecutiens*, *A*. *kurzii*, *A*. *protentum*, *A*. *laticeps*, and *A*.*murraystuarti*: Data from Ng and Wright [4], and Ng and Kottelat [9].

*A*. *tenuispinis* and *A*. *cerinum*: Data from Ng and Wright [1].

*A*. *carinatum*: Data from Ng [11].

*A*. *macropterus*: Data from Ng [12].

## Supporting Information

S1 Fig Lateral view of *Amblyceps waikhomi*, paratype, RGUMF 271, 30.4 mm SL. (TIF)

S2 Fig Caudal fin of *Amblyceps waikhomi*, holotype, ZSI/APRC/P-1125, 42.9 mm SL. (TIF)

S3 Fig Photograph of medial caudal-fin rays of *Amblyceps mangois*. Lubpr: lowermost upper branched principal ray; Sdp: strongly developed-projections (Bifid projections are artifacts arising from misplacement of the other half of the lepidotrichia during clearing and staining process)
(TIF)

S1 Table Data of total vertebrae count and body depth at anus of 19 species of *Amblyceps*. (DOCX)

S2 Table Morphometric data and gill rakers count of *Amblyceps mangois* (Hamilton). (DOCX)

S3 Table Morphometric data of *Amblyceps arunachalensis* Nath and Dey. (DOCX)

S4 Table Morphometric data of *Amblyceps apangi* Nath and Dey. (DOCX)

## References

[1] Ng HH, Wright JJ. *Amblycepscerinum*, a new catfish (Teleostei: Amblycipitidae) from northeastern India. Zootaxa. 2010; 2672: 50–60.

[2] Chen XP, Lundberg JG. *Xiurenbagrus*, a new genus of amblycipitide catfishes (Teleostei: Siluriformes), and phylogenetic relationships among the genera of Amblycipitidae. Copeia. 1995; 4: 780–800.

[3] Eschmeyer WN. [Cited 26 April 2015]. Catalog of Fishes: Genera, Species, References. Available: (http://researcharchive.calacademy.org/research/ichthyology/catalog/fishcatmain.asp).

[4] Ng HH, Wright JJ. A new torrent catfish from western Thailand (Siluriformes: Amblycipitidae). Copeia. 2009; 2: 369–377.

[5] Roberts TR. Revision of the striped catfishes of Thailand misidentified as *Mystus vittatus*, with descriptions of two new species (Pisces: Bagridae). Ichthyol. Explor. Freshw. 1992; 3: 77–88.

[6] Roberts TR. Systematic revision of Asian bagrid catfishes of the genus *Mystus* sensu stricto, with a new species from Thailand and Cambodia. Ichthyol. Explor. Freshw. 1994; 5: 241–256.

[7] Hollister G. Clearing and dying fish for bone study. Zoologica. 1934; 12: 89–101.

[8] APHA. Standard methods for the examination of water and waste water. 21st ed. Washington DC: American Public Health Association; 2005.

[9] Ng HH, Kottelat M. A review of the genus *Amblyceps* (Osteichthyes: Amblycipitidae) in Indo-china, with descriptions of five new species. Ichthyol. Explor. Freshw.2000; 11:335–348.

[10] Linthoigambi I, Vishwanath W. Two new catfish species of the genus Amblyceps from Manipur, India (Teleostei: Amblycipitidae). Ichthyol. Explor. Freshw. 2008; 19: 167–174.

[11] Ng HH. *Amblyceps carinatum*, a new species of torrent catfish from Myanmar (Teleostei: Amblycipitidae). Raffles B Zool. 2005; 53: 243–249.

[12] Ng HH. *Amblyceps macropterus*, a new species of amblycipitid catfish (Osteichthyes: Amblycipitidae) from Pakistan. Ichthyol. Explor. Freshw. 2001; 12: 201–204.

