# Supplementary figures and images for: Amblyceps waikhomi, a New Species of Catfish (Siluriformes: Amblycipitidae) from the Brahmaputra Drainage of Arunachal Pradesh, India
Source: PLoS One. 2016 Feb 3;11(2):e0147283. doi: 10.1371/journal.pone.0147283 (PMC4740403; doi:10.1371/journal.pone.0147283)

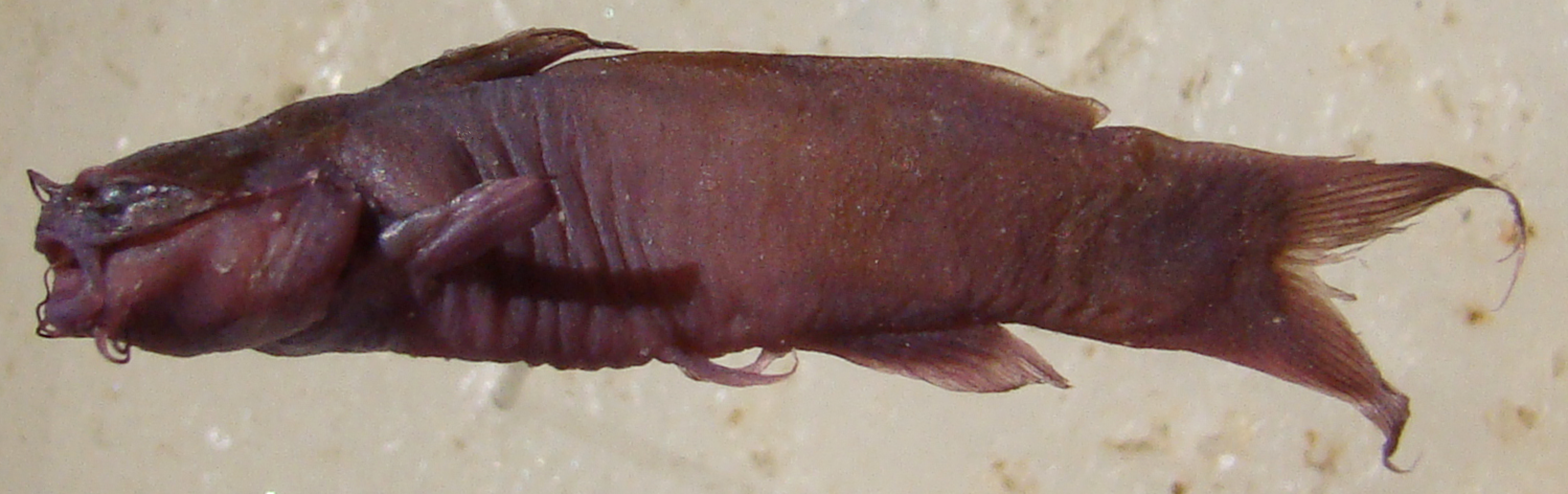

Supplement: S1 Fig — (TIF) [file pone.0147283.s001.tif]

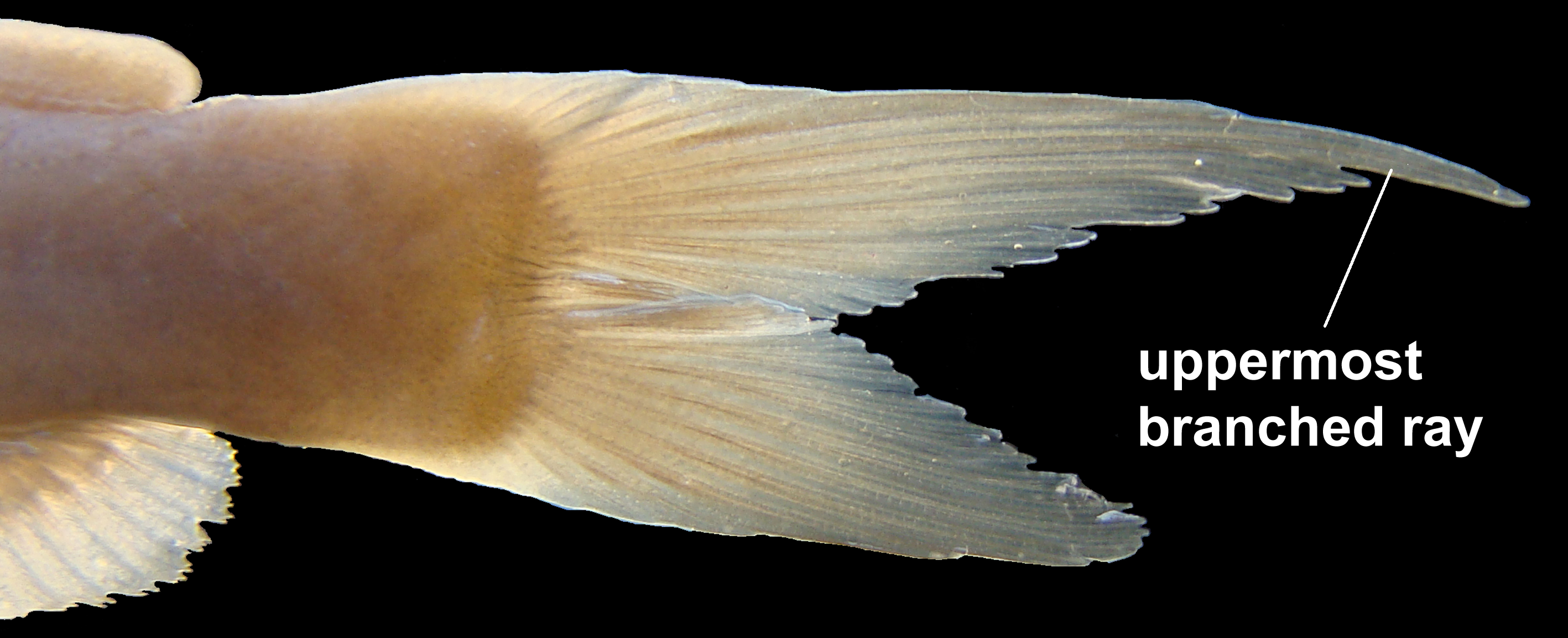

Supplement: S2 Fig — (TIF) [file pone.0147283.s002.tif]

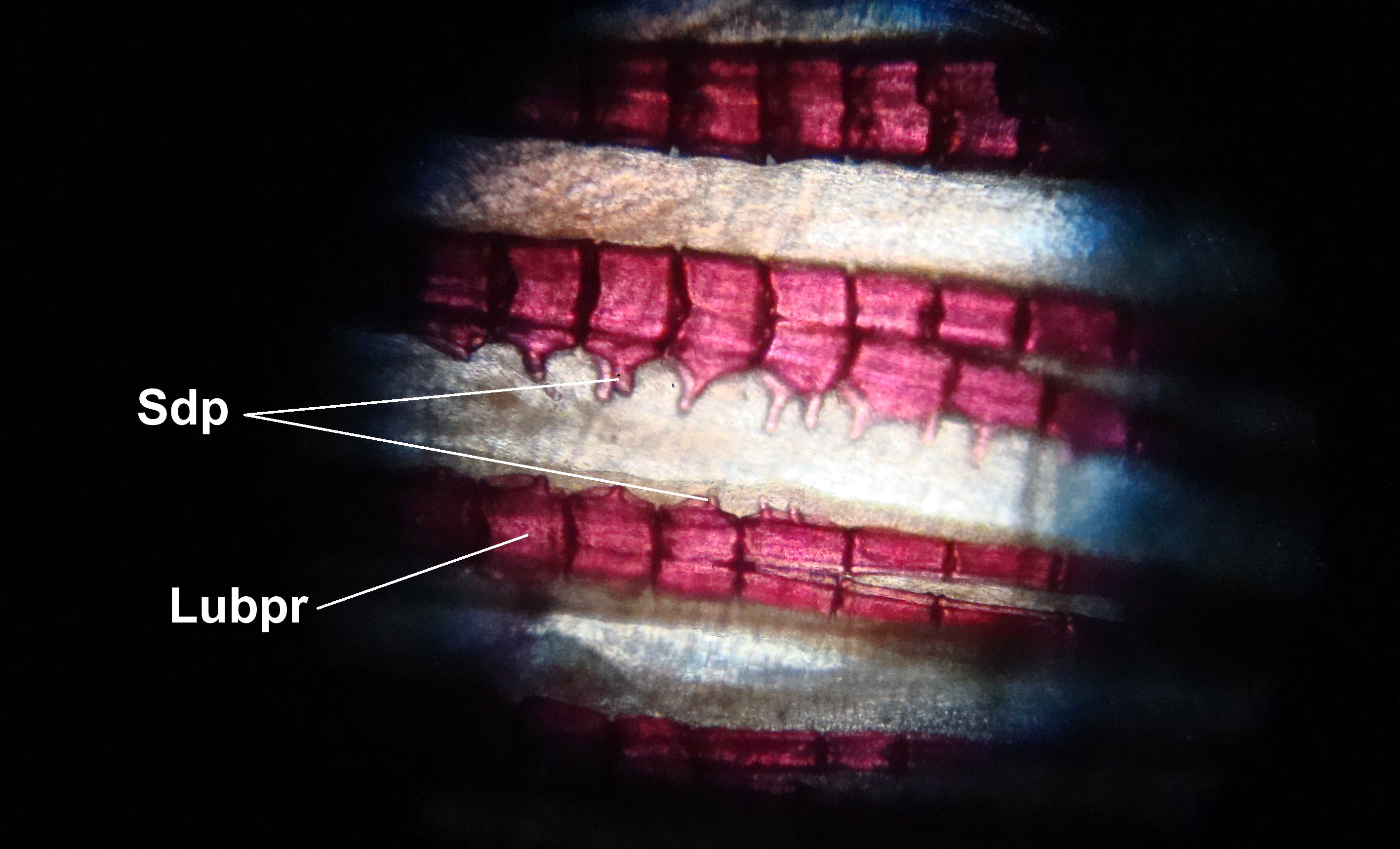

Supplement: S3 Fig — Lubpr: lowermost upper branched principal ray; Sdp: strongly developed-projections (Bifid projections are artifacts arising from misplacement of the other half of the lepidotrichia during clearing and staining process) (TIF) [file pone.0147283.s003.tif]
