# Supplementary material for: Amblyceps waikhomi, a New Species of Catfish (Siluriformes: Amblycipitidae) from the Brahmaputra Drainage of Arunachal Pradesh, India
Source: PLoS One. 2016 Feb 3;11(2):e0147283. doi: 10.1371/journal.pone.0147283 (PMC4740403; doi:10.1371/journal.pone.0147283)
Supplement: S1 Table — (DOCX) [file pone.0147283.s004.docx]

**S1 Table. Data of total vertebrae count and body depth at anus of 19 species of *Amblyceps*.**

| Sl. No. | Species | Total vertebrae count | Body depth at anus (in % SL) |
| --- | --- | --- | --- |
|  | *Amblyceps waikhomi* | 34–35 | 17.0–20.3 |
|  | *A. mangois* (Hamilton) | 34–36 | 12.5–15.8 |
|  | *A. arunachalensis* Nath and Dey | 38 | 10.1–11.1 |
|  | *A. tenuispinis* Blyth | 37–38 [1] | 9.2–12.8 [1] |
|  | *A. apangi* Nath and Dey | 48 | 10.2–12.1 |
|  | *A. laticeps* (McClelland) | 41–43 [4] | 7.6–11.1 [9] |
|  | *A. cerinum* Ng and Wright | 44–48* [1] | 9.2–11.9 [1] |
|  | *A*. *macropterus* Ng | 37 [12] | 11.5 [12] |
|  | *A.* *torrentis* Linthoingambi and Vishwanath | 46–47 [10] | 15.3–17.3 |
|  | *A.* *tuberculatum* Linthoingambi and Vishwanath | 40 [10] | 13.6–16.8 |
|  | *A. caecutiens* Blyth | 40# [9] | 12.8–15.1# [9] |
|  | *A.* *protentum* Ng and Wright | 41–42 [4] | 8.0–11.0 [4] |
|  | *A.* *carinatum* Ng | 39–40 [11] | 13.1–15.0 [11] |
|  | *A.* *murraystuarti* Chaudhuri | 45–46 [4] | 11.6–15.0 [9] |
|  | *A.* *platycephalus* Ng and Kottelat | 41 [9] | 11.5 [9] |
|  | *A.* *variegatum* Ng and Kottelat | 39–41 [9] | 12.6–14.1 [9] |
|  | *A.* *foratum* Ng and Kottelat | 38–41 [9] | 11.2–14.2 [9] |
|  | *A.* *serratum* Ng and Kottelat | 34–36 [4] | 15.2–16.9 [9] |
|  | *A. kurzii* (Day) | 40–41 [9] | 9.7–10.5 [4] |

*1(first centrum) + 3 (complex centrum) + 41–44 (post-Weberian vertebrae) = 44–48

# Data of *A. mucronatum* which is a junior synonym of *A. caecutiens*
