# Supplementary material for: Amblyceps waikhomi, a New Species of Catfish (Siluriformes: Amblycipitidae) from the Brahmaputra Drainage of Arunachal Pradesh, India
Source: PLoS One. 2016 Feb 3;11(2):e0147283. doi: 10.1371/journal.pone.0147283 (PMC4740403; doi:10.1371/journal.pone.0147283)
Supplement: S2 Table — (DOCX) [file pone.0147283.s005.docx]

**S2 Table. Morphometric data and gill rakers count of *Amblyceps mangois* (Hamilton).**

| Specimens catalogue number and collection sites | ZSI-NRS/F2556 (Padhoe river) & RGMF (uncat.) (Gola River) | MUMF 14301-14302  (Jiri River) | MUMF 14061 (Dikrong River) | Range |
| --- | --- | --- | --- | --- |
| Basin | Ganga | Barak | Brahmaputra |  |
| Standard length | 40.0– 55.5 | 35.7–37.1 | 48.0 | 35.7– 55.5 |
| In % SL | | | | |
| Predorsal length | 28.6–33.1 | 29.7–29.9 | 29.5 | 28.6–33.1 |
| Preanal length | 62.1–65.7 | 64.1–66.3 | 64.3 | 62.1–66.3 |
| Prepelvic length | 45.3–47.7 | 46.4–52.2 | 49.3 | 45.3–52.2 |
| Prepectoral length | 16.8–19.7 | 15.6–20.7 | 19.5 | 15.6–20.7 |
| Length of anal-fin base | 13.4–18.0 | 14.4–14.8 | 18.1 | 13.4–18.1 |
| Pelvic-fin length | 10.9–13.2 | 10.6–10.8 | 12.0 | 10.6–13.2 |
| Pectoral-fin length | 14.2–17.7 | 15.6–17.9 | 16.0 | 14.2–17.9 |
| Upper lobe of Caudal-fin length | 27.9–38.4 | 29.6–30.4 | 32.2 | 27.9–38.4 |
| Adipose-fin base length | 16.9–24.0 | 22.3–25.2 | 21.5 | 16.9–25.2 |
| Dorsal to adipose-fin length | 19.1–20.5 | 21.0–22.4 | 23.2 | 19.1–22.4 |
| Length of caudal peduncle | 15.8–20.8 | 18.6–19.3 | 18.5 | 15.8–20.8 |
| Depth of caudal peduncle | 11.7–17.9 | 12.9–16.2 | 9.9 | 9.9–17.9 |
| Body depth at anus | 12.5–15.8 | 15.6–15.7 | 15.4 | 12.5–15.8 |
| Head length | 18.8–20.2 | 21.0–21.3 | 21.0 | 18.8–21.3 |
| Head width | 17.1–18.3 | 17.6–18.3 | 17.7 | 17.1–18.3 |
| Head depth at occiput | 11.6–12.1 | 12.0–13.2 | 12.7 | 11.6–13.2 |
| In % HL | | | | |
| Snout | 24.7–30.0 | 32.8–38.4 | 41.5 | 24.7–41.5 |
| Eye diameter | 6.9–10.1 | 9.2–10.2 | 5.9 | 6.9–10.2 |
| Inter orbital distance | 33.0–41.8 | 40.7–42.3 | 38.6 | 33–42.3 |
| Nasal barbel length | 81.4–101.3 | 75.6–86.4 | 61.3 | 61.3–101.3 |
| Maxillary barbel length | 96.2–128 | 94.7–119.2 | damage | 94.7–119.2 |
| Inner mandibular barbel length | 67.0–94.9 | 56.5–74.3 | 74.2 | 56.5–94.9 |
| Outer mandibular barbel length | 87.2–116.4 | 80.7–86.8 | 85.1 | 80.7–116.4 |
| Gill rakers count | 1+9=10 (n=3) | 1+9=10 (n=2) | 1+9=10 | 10 |
