# Supplementary material for: Amblyceps waikhomi, a New Species of Catfish (Siluriformes: Amblycipitidae) from the Brahmaputra Drainage of Arunachal Pradesh, India
Source: PLoS One. 2016 Feb 3;11(2):e0147283. doi: 10.1371/journal.pone.0147283 (PMC4740403; doi:10.1371/journal.pone.0147283)
Supplement: S3 Table — (DOCX) [file pone.0147283.s006.docx]

| RGUMF 117 (n=3) | | | | |
| --- | --- | --- | --- | --- |
|  |  | | | Range |
| Total length | 114.6 | 111.0 | 121.1 | 111.0–114.6 mm |
| Standard length | 85.1 | 82.6 | 97.2 | 82.6–97.2 mm |
| In % SL |  |  |  |  |
| Predorsal length | 23.2 | 22.5 | 22.8 | 22.5–23.2 |
| Preanal length | 63.0 | 64.1 | 62.2 | 62.2–64.1 |
| Prepelvic length | 45.1 | 45.7 | 45.9 | 45.1–45.9 |
| Prepectoral length | 19.8 | 19.3 | 18.2 | 18.2–19.8 |
| Length of dorsal-fin base | 11.6 | 11.8 | 11.7 | 11.6–11.8 |
| Length of anal-fin base | 17.3 | 17.0 | 16.4 | 16.4–17.3 |
| Pelvic- fin length | 9.1 | 8.5 | 8.7 | 8.5–9.1 |
| Pectoral-fin length | 13.8 | 13.8 | 12.8 | 12.8–13.8 |
| Upper lobe of Caudal-fin length | 31.4 | 32.5 | Damage | 31.4–32.5 |
| Lower lobe of Caudal-fin length | 22.0 | 22.8 | Damage | 22.0–22.8 |
| Length of adipose-fin base | 19.0 | 19.8 | 18.1 | 18.1–19.8 |
| Dorsal to adipose distance | 31.8 | 30.7 | 30.1 | 30.1–31.8 |
| Post adipose distance | 15.9 | - | 16.6 | 15.9–16.6 |
| Length of caudal peduncle | 19.3 | 19.1 | 19.6 | 19.1–19.6 |
| Depth of caudal peduncle | 9.2 | 9.1 | 8.2 | 8.2–9.2 |
| Body depth at anus | 11.0 | 11.1 | 10.0 | 10.0–11.1 |
| Head length | 18.4 | 19.0 | 17.2 | 17.2–19.0 |
| Head width | 15.0 | 14.8 | 13.6 | 13.6–15.0 |
| Head depth at occiput | 10.7 | 11.0 | 11.1 | 10.7–11.1 |
| % HL |  |  |  |  |
| Snout | 29.9 | 29.2 | 28.7 | 28.0–29.2 |
| Eye diameter | 6.3 | 5.7 | 6.5 | 5.7–6.5 |
| Inter orbital distance | 37.5 | 35.0 | 34.7 | 34.7–37.5 |
| Nasal barbel length | 74.5 | 68.7 | 68.2 | 68.2–74.5 |
| Maxillary barbel length | 94.2 | 98.7 | 92.2 | 92.2–98.7 |
| Inner mandibular barbel length | 57.9 | 42.6 | 50.2 | 42.6–57.9 |
| Outer mandibular barbel length | 91.0 | 63.6 | 76.6 | 63.6–91.0 |

**S3 Table. Morphometric data of *Amblyceps arunachalensis* Nath and Dey.**
