# Supplementary material for: Amblyceps waikhomi, a New Species of Catfish (Siluriformes: Amblycipitidae) from the Brahmaputra Drainage of Arunachal Pradesh, India
Source: PLoS One. 2016 Feb 3;11(2):e0147283. doi: 10.1371/journal.pone.0147283 (PMC4740403; doi:10.1371/journal.pone.0147283)
Supplement: S4 Table — (DOCX) [file pone.0147283.s007.docx]

|  | RGUMF  118 | RGUMF  116 | | | | | | | RGUMF  114 | | Range |
| --- | --- | --- | --- | --- | --- | --- | --- | --- | --- | --- | --- |
| Standard length | 160 | 71.5 | 74.4 | 75.7 | 85.4 | 102.5 | 112.3 | 120.0 | 81.8 | 91.2 |  |
| In % SL |  |  |  |  |  |  |  |  |  |  |  |
| Predorsal length | 27.4 | 25.8 | 25.3 | 26.0 | 23.7 | 24.5 | 23.5 | 22.2 | 23.1 | 22.6 | 22.2–27.4 |
| Preanal length | 65.3 | 68.5 | 65.3 | 64.6 | 66.0 | 65.2 | 66.9 | 62.8 | 65.8 | 67.2 | 62.8–68.5 |
| Prepelvic length | 46.6 | 45.5 | 47.7 | 45.2 | 49.3 | 46.7 | 48.5 | 45.2 | 46.4 | 46.7 | 45.2–49.3 |
| Prepectoral length | 18.0 | 16.6 | 18.3 | 18.2 | 14.2 | 19.3 | 20.8 | 18.8 | 18.9 | 18.5 | 14.2–20.8 |
| Length of dorsal - fin base | 5.9 | 7.3 | 6.2 | 6.7 | 6.1 | 6.7 | 6.1 | 6.3 | 7.7 | 7.8 | 5.9–7.8 |
| Length of anal-fin base | 9.8 | 12.0 | 10.1 | 12.2 | 11.3 | 11.8 | 11.0 | 12.6 | 13.3 | 11.8 | 9.8–13.3 |
| Pelvic- fin length | 8.1 | 8.1 | 10.2 | 9.4 | 9.1 | 7.5 | 8.5 | 7.6 | 6.7 | 8.7 | 6.7–10.2 |
| Pectoral-fin length | 10.9 | 12.8 | 14.1 | 14.0 | 12.6 | 12.7 | 12.0 | 11.6 | 12.7 | 11.3 | 10.9–14.1 |
| Length of adipose-fin base | 27.8 | 27.0 | 22.0 | 28.7 | 26.6 | 28.1 | 28.5 | 26.3 | 30.8 | 32.6 | 22.0–32.6 |
| Dorsal to adipose distance | 35.7 | 31.4 | 34.8 | 29.2 | 34.5 | 32.0 | 31.5 | 31.8 | 35.6 | 29.9 | 29.2–35.7 |
| Post adipose distance | 8.3 | 8.1 | 7.8 | 8,0 | 7.0 | 7.6 | 9.0 | 8.5 | 9.0 | 9.8 | 7.0–9.8 |
| Body depth at anus | 10.6 | 11.9 | 11.8 | 10.2 | 12.1 | 11.1 | 10.2 | 10.7 | 11.2 | 10.7 | 10.2–12.1 |
| Head length | 25.3 | 23.1 | 19.4 | 20.1 | 23.2 | 20.9 | 21.6 | 19.5 | 20.5 | 19.2 | 19.2–25.3 |
| Head width | 18.3 | 15.7 | 14.9 | 12.9 | 14.9 | 16.1 | 16.1 | 13.5 | 15.4 | 14.4 | 12.9–18.3 |
| Head depth at occiput | 9.8 | 9.1 | 9.4 | 8.3 | 9.8 | 10.4 | 10.0 | 9.3 | 9.7 | 9.8 | 8.3–10.4 |
| In % HL |  |  |  |  |  |  |  |  |  |  |  |
| Snout | 39.6 | 30.9 | 39.3 | 38.8 | 36.9 | 38.6 | 33.7 | 29.1 | 33.3 | 32.3 | 29.1–39.6 |
| Inter orbital distance | 22.9 | 22.4 | 26.2 | 25.7 | 26.7 | 21.9 | 24.2 | 25.2 | 26.8 | 25.0 | 21.9–26.93 |
| Maxillary barbel length | 77.8 | 85.5 | 86.2 | 107.2 | 85.4 | 79.5 | 78.2 | 75.6 | 102.3 | 97.7 | 75.6–105.5 |

**S4 Table. Morphometric data *of Amblyceps apangi* Nath and Dey (n=10).**
